# Tacrolimus-Induced Thrombotic Microangiopathy After Orthotopic Heart Transplant: A Case Report

**DOI:** 10.7759/cureus.25874

**Published:** 2022-06-12

**Authors:** Rajwinder Gill, Vineet Meghrajani

**Affiliations:** 1 Internal Medicine, Icahn School of Medicine at Mount Sinai Beth Israel, New York, USA; 2 Cardiovascular Medicine, Icahn School of Medicine at Mount Sinai Beth Israel, New York, USA

**Keywords:** heart transplant, hemolytic uremic syndrome, hus, acquired ttp, ttp, drug induced thrombotic microangiopathy, thrombotic microangiopathy (tma), tma, tacrolimus, ditma

## Abstract

Tacrolimus (FK 506) is a calcineurin inhibitor and is commonly used as an immunosuppressant after a solid organ transplant. One of the serious adverse effects of tacrolimus is thrombotic microangiopathy (TMA) which has a high mortality rate. TMA leads to vascular thrombosis, eventually leading to ischemia of end organs. It is diagnosed clinically and based on the laboratory parameters. Early detection of TMA and prompt treatment can change the course. Drug-induced TMA has a poor prognosis as compared to idiopathic TMA.

We present here the case of a 47-year-old male who developed tacrolimus-induced TMA after an orthotopic heart transplant and he was treated with the currently available treatment. He ultimately died after 40 days of hospitalization.

## Introduction

Thrombotic microangiopathy (TMA) is an infrequent but severe complication of tacrolimus. Following the endothelial injury, it leads to the formation of microthrombi with platelet aggregation in the vasculature. The microthrombi result in the mechanical destruction of erythrocytes known as microangiopathic hemolytic anemia and fragments of erythrocytes known as schistocytes are seen on a peripheral blood smear. Laboratory parameters are consistent with hemolysis, i.e., decreased hemoglobin, decreased haptoglobin, increased lactate dehydrogenase (LDH), increased reticulocyte count, unconjugated hyperbilirubinemia, and thrombocytopenia are seen due to platelet aggregation. Transient decrease in ADAMTS-13 (a disintegrin and metalloproteinase with thrombospondin type 1 motif, member 13) is seen in all forms of TMA. The clinical outcomes are due to ischemic injury and are dependent on vasculature involvement: acute renal failure due to renal vasculature involvement known as a hemolytic uremic syndrome (HUS) and predominant neurologic involvement known as thrombotic thrombocytopenic purpura (TTP) [1-3].

## Case presentation

A 47-year-old male with a history of ventricular fibrillation arrest s/p automated implantable cardiac defibrillator (AICD) in 2018, cerebrovascular accident in 2018 with residual cognitive and speech impairment s/p patent foramen ovale repair, coronary artery disease s/p coronary artery bypass grafting, heart failure with reduced ejection fraction [EF] s/p left ventricular assist device (LVAD), diabetes mellitus, chronic kidney disease stage 3 (CKD 3), recurrent atrial arrhythmias came for allograft orthotopic heart transplant. An orthotopic heart transplant was done along with LVAD explantation and AICD removal without any complications. He was transfused five units of packed red blood cells, one unit of fresh frozen plasma, and one unit of platelets during the surgery. He was transferred to the intensive care unit (ICU) after surgery. 

On arrival at ICU, he was intubated and was requiring four vasopressors for blood pressure support and inotropic support. He continued to be in cardiogenic shock likely due to acute allograft dysfunction and vasoplegic on a high dose of vasopressors. On day 1 post-surgery, lactate was trending up showing decreased perfusion to end organs and transthoracic echocardiogram (TTE) showed akinetic right ventricle (RV) and severely hypokinetic left ventricle (LV). Following this, veno-arterial extracorporeal membrane oxygenation (VA-ECMO) was emergently placed. His urine output was low and continuous veno-venous hemofiltration was started due to volume overload. As per the medications, he was started on vancomycin, ceftazidime and avibactam, and aztreonam as surgical tissue culture showed gram-positive cocci in pairs. These antibiotics were chosen given his history of multi-drug-resistant organisms. As per his post-transplant medications, he was on solumedrol taper and intravenous mycophenolate mofetil post-surgery. Tacrolimus (FK 506) was started on day 3 after surgery and was titrated based on the blood level. He was also started on valacyclovir, trimethoprim-sulfamethoxazole, and caspofungin on day 3 as he was on immunosuppressants. 

**Figure 1:**
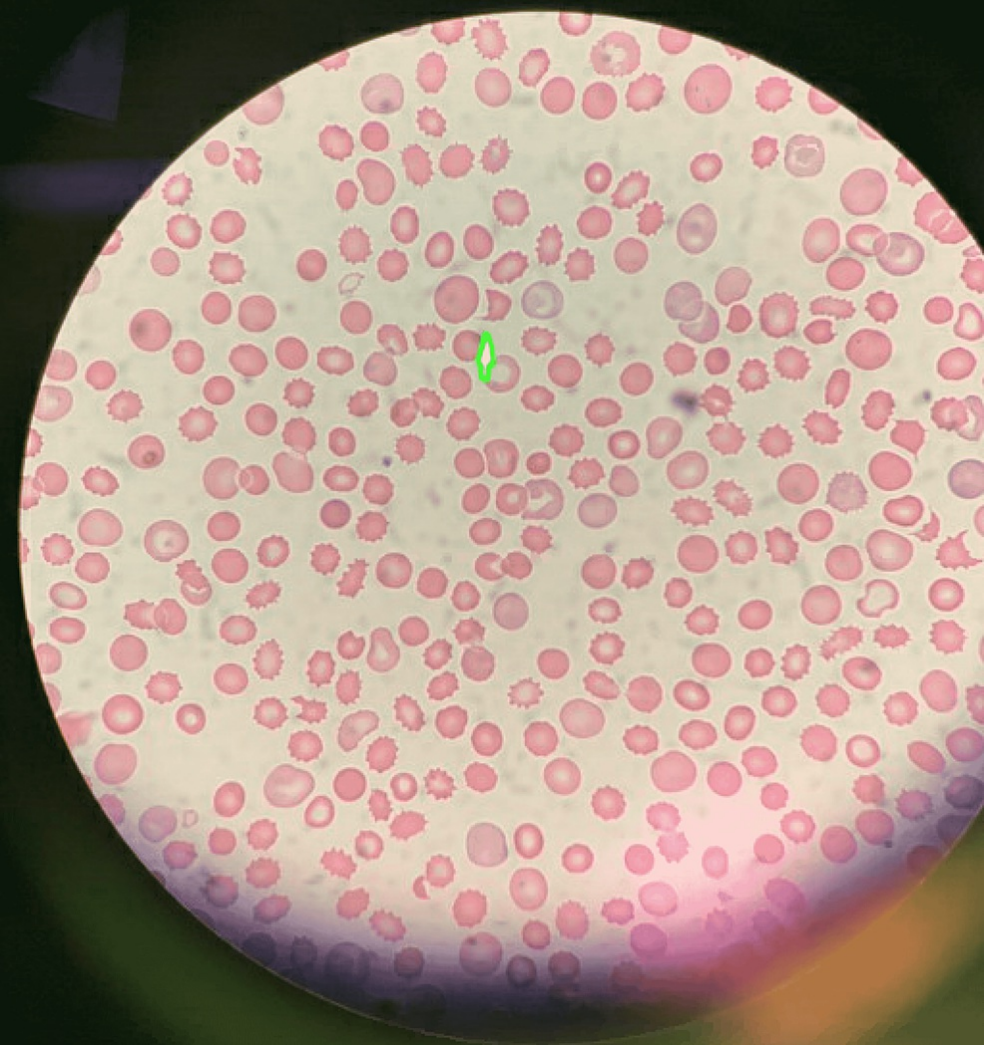
Peripheral blood smear showing schistocytes

**Figure 2:**
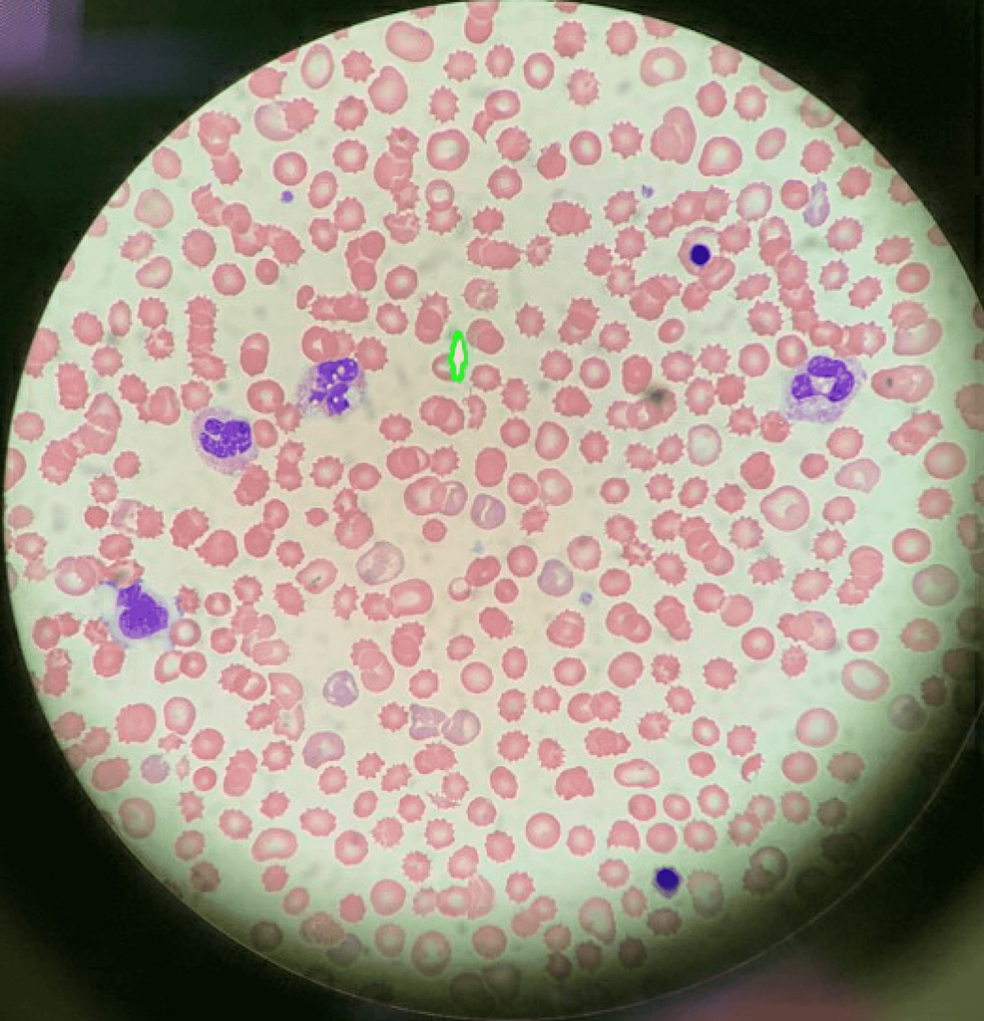
Peripheral blood smear showing multiple burr cells as a response to profound stress

On day 7, TTE showed the improvement in LV function with an EF of 53% but RV was severely akinetic. He was also started on methylene blue infusion to help with vasoplegia without any improvement. He started developing bullous skin lesions on his extremities along with skin mottling and a skin biopsy was done in concern for Steven Johnson syndrome (SJS). His labs on day 7 showed thrombocytopenia, low haptoglobin, and elevated LDH. A peripheral blood smear showed schistocytes and multiple burr cells displayed in Figures 1, 2. We presumed these findings were likely due to hemolysis and thrombocytopenia likely due to consumption as he was on VA-ECMO but we checked the ADAMTS13 along with the antibodies for heparin-induced thrombocytopenia (HIT) and anti-globulin antibodies. VA-ECMO was decannulated and a percutaneous right ventricular assist device (RVAD) and an intra-aortic balloon pump (IABP) were placed. He required multiple platelet transfusions due to thrombocytopenia. We stopped valacyclovir and started atovaquone given the thrombocytopenia. 

**Figure 3:**
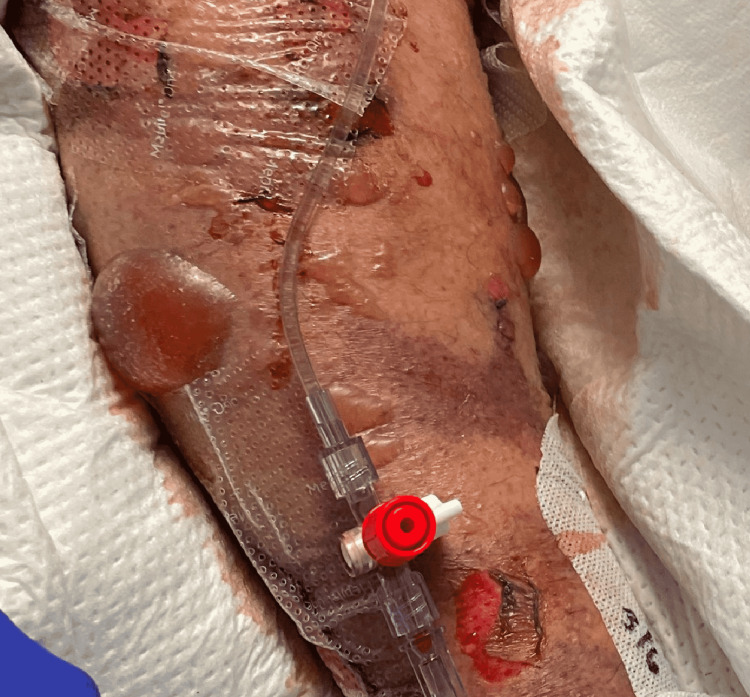
Bullous skin lesions and mottling of skin on arm

On day 12 post-surgery, he had worsening skin mottling with involvement around the eye and some desquamating lesions as well which was very suspicious for SJS as shown in Figure 3. So we started on the three-day course of intravenous immunoglobulin after obtaining normal IgA levels. We stopped aztreonam and ceftazidime-avibactam. On day 13, a skin biopsy resulted and a frozen section of the skin showed thrombi arousing the concern for drug-induced thrombotic microangiopathy (DITMA). Tests for viral infections, i.e. cytomegalovirus, influenza A, human immunodeficiency virus (HIV), parvovirus B-19, BK polyoma virus, and human herpes virus-6 were sent, which resulted negative. We stopped tacrolimus and started him on belatacept. On day 14, plasma exchange (PLEX) therapy started. He was still requiring four vasopressors. A three-day course of pulse dose steroids was started on day 14 following which he was transitioned to the initial dosage. On day 15, ADAMTS13 activity resulted in 47% (normal) and HIT antibodies and anti-globulin resulted negative following which PLEX was stopped after one session. IABP was removed on day 15. On day 18, skin biopsy for complement staining showed fibrin deposits and complement suggestive of antiphospholipid syndrome (APLS) versus atypical hemolytic uremic syndrome (aHUS). It detected pauci inflammatory TMA with abundant complement C5-9 deposition. We started eculizumab weekly after giving meningococcal vaccines along with levofloxacin prophylaxis. He was started on a bivalirudin drip after biopsy results were suggestive of APLS and a hypercoagulable workup was sent which resulted negative. A complement panel for the aHUS was sent which resulted negative. 

He still required four vasopressors, and inhaled nitric oxide (iNO) was started to wean off the RVAD flow. Hemolysis labs showed improvement with a decrease in LDH as well as bilirubin and an increase in haptoglobin, platelet count, and decreased schistocytes on peripheral smear. Skin lesions also started improving along with decreased hemolysis. There was no improvement in renal function after four doses of eculizumab. An echocardiogram was done weekly, which showed normal left ventricular function and improvement of right ventricular function as well. We were able to wean him off the RVAD by day 25 with iNO. His vasopressor requirements decreased and we were able to wean off one of the four vasopressors. On day 34, he started requiring higher doses of vasopressors and laboratory workup started showing increased lactate. He was unresponsive and was not following commands. A family meeting was done and after looking at his suffering, the family opted for comfort care. The patient died on day 41 of hospitalization. The trend of all the laboratory workups is shown in Table 1. 

**Table 1:** Laboratory workup trended through the hospital course

Test	Day 1	Day 7	Day 14	Day 30	Day 40	Reference Range
White blood count (WBC)	9.6	25.6	13.0	12.3	13.7	4.5-11.00 k/uL
Hemoglobin	11.6	9.3	8.4	8.9	6.9	13.6-16.3 g/dL
Platelet	268	30	64	68	37	150-450 k/uL
Sodium	136	135	134	135	133	135-145 meq/L
Potassium	4.3	4.4	4.5	4.3	4.7	3.5-5.2 mmol/L
Chloride	99	101	103	102	101	96-108 mmol/L
Phosphorus	2.6	2.7	2.4	2.8	2.9	2.4-4.7 mg/dL
Magnesium	2.2	2.1	2.4	2.0	2.1	1.5-2.5 mg/dL
Creatinine	1.76	3.60	2.62	1.52	0.61	0.5-1.1 mg/dL
Blood urea nitrogen	28	36	55	37	35	6-23 mg/dL
Aspartate aminotransferase	179	1275	140	78	95	1-35 U/L
Alanine aminotransferase	32	657	81	83	99	1-45 U/L
Alkaline phosphatase	64	183	97	75	190	38-126 U/L
Total bilirubin	1.3	2.9	7.6	2.0	3.0	0.1-1.2 mg/dL
pH	7.30	7.31	7.36	7.37	7.23	7.35-7.45
pCO_2_	36	39	37	36	37	35-45 mmHg
Bicarbonate	17.4	17.5	20.3	22.7	19.7	21-29 mEq/L
Lactic acid	>15	3.2	3.1	1.8	6.8	0.50-2.00 mmol/L
Haptoglobin	NA	<8	<8	45	36	30-200 mg/dL
Lactate dehydrogenase (LDH)	NA	2992	1584	508	749	100-220 U/L
Reticulocyte count	NA	1.7	11.2	10.8	9.4	0.7-2.8%
INR (international normalized ratio)	1.6	1.4	1.5	1.6	1.8	1.0-1.5
Prothrombin time (PT)	18.1	17.9	18.2	16.8	19.7	12.3-14.9 seconds
Partial thromboplastin time (PTT)	127	38	32	58.5	60.5	25.4-34.9 seconds

He remained vasoplegic throughout the hospital course due to unclear reasons. His decline near to demise was likely due to sepsis without any improvement on broad-spectrum antibiotics.

## Discussion

Etiology and pathogenesis**

The etiology of TMA can be genetic (ADAMTS-13 deficit and coagulation pathway mutations), bacterial and viral infections, malignancy, pregnancy, autoimmune diseases, HIV, and certain medications like calcineurin inhibitors (CNI), bevacizumab, mitomycin C, etc [4]. DITMA associated with tacrolimus has reported incidence ranging from 1% to 4.7% as per current literature [5]. 

Most of the cases reported having tacrolimus-induced TMA have been post-renal transplants. The pathogenesis of calcineurin inhibitors that induce TMA is not clear. It is believed to be due to direct endothelial injury. Albeit the mechanism of direct endothelial injury is not clear, it is implied to be immune-mediated or dose-dependent. Clinical features of immune-mediated are acute and dose-dependent are typically gradual [6,7]. It is not related to the trough levels of tacrolimus. 

Diagnosis and treatment 

Diagnosis of TMA is clinicopathological. The constellation of anemia, thrombocytopenia, and presence of schistocytes on a peripheral blood smear is enough to start treatment given that mortality is very high. Other laboratory markers are decreased haptoglobin, increased LDH, increased reticulocytes, and unconjugated hyperbilirubinemia. ADAMTS-13 activity should be checked and collected before starting the therapy. The direct antiglobulin test is negative and the coagulation profile is usually normal but can be abnormal in severe cases with disseminated intravascular coagulopathy [3]. If ADAMTS-13 is normal, the complement panel for aHUS should be tested next [8]. In post-transplant patients, viral causes of TMA should be tested before concluding it as DITMA. 

Once TMA is suspected, PLEX should be started promptly and the offending drug should be stopped. Plasma infusion therapy can be started if PLEX is not available right away [9]. Corticosteroids are also added as part of the initial therapy [10]. Eculizumab should be considered in patients not responding to PLEX and with normal ADAMTS-13 activity [11,12]. The prognosis after DITMA has been seen to be poor post-transplant patients [5].

## Conclusions

Although clinical and laboratory findings are similar in idiopathic TTP/HUS and DITMA, response to current treatment and prognosis is very poor in DITMA. Low ADAMTS-13 activity as found in idiopathic TTS/HUS is found to be normal in post-transplant patients. ADAMTS-13 activity in post-transplant patients is still controversial. Advances in treatment modalities are needed to enhance the outcomes in DITMA.

## References

[1] Qu L, Kiss JE (2005). Thrombotic microangiopathy in transplantation and malignancy. Semin Thromb Hemost.

[2] Nwaba A, MacQuillan G, Adams LA (2013). Tacrolimus-induced thrombotic microangiopathy in orthotopic liver transplant patients: case series of four patients. Intern Med J.

[3] Arnold DM, Patriquin CJ, Nazy I (2017). Thrombotic microangiopathies: a general approach to diagnosis and management. CMAJ.

[4] Bommer M, Wölfle-Guter M, Bohl S, Kuchenbauer F (2018). The differential diagnosis and treatment of thrombotic microangiopathies. Dtsch Arztebl Int.

[5] Pisoni R, Ruggenenti P, Remuzzi G (2001). Drug-induced thrombotic microangiopathy: incidence, prevention and management. Drug Saf.

[6] Chatzikonstantinou T, Gavriilaki M, Anagnostopoulos A, Gavriilaki E (2020). An update in drug-induced thrombotic microangiopathy. Front Med (Lausanne).

[7] Al-Nouri ZL, Reese JA, Terrell DR, Vesely SK, George JN (2015). Drug-induced thrombotic microangiopathy: a systematic review of published reports. Blood.

[8] Bu F, Maga T, Meyer NC, Wang K, Thomas CP, Nester CM, Smith RJ (2014). Comprehensive genetic analysis of complement and coagulation genes in atypical hemolytic uremic syndrome. J Am Soc Nephrol.

[9] Rock GA, Shumak KH, Buskard NA, Blanchette VS, Kelton JG, Nair RC, Spasoff RA (1991). Comparison of plasma exchange with plasma infusion in the treatment of thrombotic thrombocytopenic purpura. Canadian Apheresis Study Group. N Engl J Med.

[10] George JN (2010). How I treat patients with thrombotic thrombocytopenic purpura: 2010. Blood.

[11] Legendre CM, Licht C, Muus P (2013). Terminal complement inhibitor eculizumab in atypical hemolytic-uremic syndrome. N Engl J Med.

[12] Gabr JB, Bilal H, Mirchia K, Perl A (2020). The use of eculizumab in tacrolimus-induced thrombotic microangiopathy. J Investig Med High Impact Case Rep.

